# 16S rRNA Gene Amplicon Sequencing Data of Tailing and Nontailing Rhizosphere Soils of Mimosa pudica from a Heavy Metal-Contaminated Ex-Tin Mining Area

**DOI:** 10.1128/MRA.00761-20

**Published:** 2020-10-15

**Authors:** Saidu Abdullahi, Hazzeman Haris, Kamarul Z. Zarkasi, Hamzah G. Amir

**Affiliations:** a School of Biological Sciences, Universiti Sains Malaysia, Pulau Pinang, Malaysia; b Department of Botany, Ahmadu Bello University, Zaria, Nigeria; University of Southern California

## Abstract

The 16S rRNA gene amplicon sequence data from tailing and nontailing rhizosphere soils of Mimosa pudica from a heavy metal-contaminated area are reported here. Diverse bacterial taxa were represented in the results, and the most dominant phyla were *Proteobacteria* (41.2%), *Acidobacteria* (17.1%), and *Actinobacteria* (14.4%).

## ANNOUNCEMENT

The rhizosphere of plants is normally colonized by microbial communities which potentially benefit the host plants (1–3). Knowledge of the bacterial community of the metal-tolerant and leguminous Mimosa pudica (found abundantly in the studied heavy metal-contaminated soil) would explain their involvement in enhancing the growth and survival of the plant (4). Studies on microbial communities from different environments provide novel insights into their structure and function, leading to the discovery of novel functional genes (5, 6). The 16S rRNA gene amplicon sequence data presented here provide insight toward understanding how the bacterial diversity impacts plant growth promotion and heavy metal tolerance.

The rhizosphere soils of *M. pudica* were collected from six different sites (tailing sites, MRS1, MRS2, and MRS3; nontailing sites, MRS4, MRS5, and MRS6) of an ex-tin mining area (latitude 5°38′N, longitude 101°1′E) in Perak, Malaysia. Physicochemical analysis (7) indicated the soils to have acidic pH (4.90 to 5.75) and to be sandy loam in nature and salt free (electrical conductivity [EC] range, 0.12 to 0.79 mS/cm). Heavy metal analysis by inductively coupled plasma optical emission spectrometry (8) detected arsenic (14.3 to 1,242 mg/kg), cadmium (1.6 to 17.8 mg/kg), and lead (52.17 to 81.10 mg/kg). *M. pudica* roots were shaken gently to remove rhizosphere soil adhering to the surface of the roots. This was done in a laminar flow cabinet, and sterile forceps were used to remove soil tightly attached to the roots (9, 10). DNA was extracted from the rhizosphere soils using a NucleoSpin soil DNA extraction kit (Macherey-Nagel, Germany) according to the manufacturer’s instructions. PCR amplification of the V3/V4 hypervariable regions was performed using the recommended Illumina 16S rRNA gene amplicon library method (USA) (11) with the primers Bakt_341F (CCTACGGGNGGCWGCAG) and Bakt_805R (GACTACHVGGGTATCTAATCC) (12). The libraries were sequenced using the MiSeq platform (Illumina).

Adapter sequences and low-quality reads were removed from the paired-end reads using BBDuk from the BBTools package (https://sourceforge.net/projects/bbmap/) (13), and forward and reverse reads were merged using USEARCH v11.0.667 (14, 15). Default parameters were used for all software unless otherwise specified. All sequences shorter than 150 bp or longer than 600 bp were removed from downstream processing. The reads were aligned with the SILVA 16S rRNA gene database (release 132) (16), inspected for chimeric errors using VSEARCH v2.6.2 (17), and then clustered *de novo* into operational taxonomic units (OTUs) at 97% similarity using UPARSE v11.0.667 (18). The taxonomic assignment of the OTUs was achieved using QIIME v1.9.1 (19–21) against the SILVA database (16). All statistical analyses were done in the R statistical package v3.6.1 (https://www.r-project.org/) (22).

**FIG 1:**
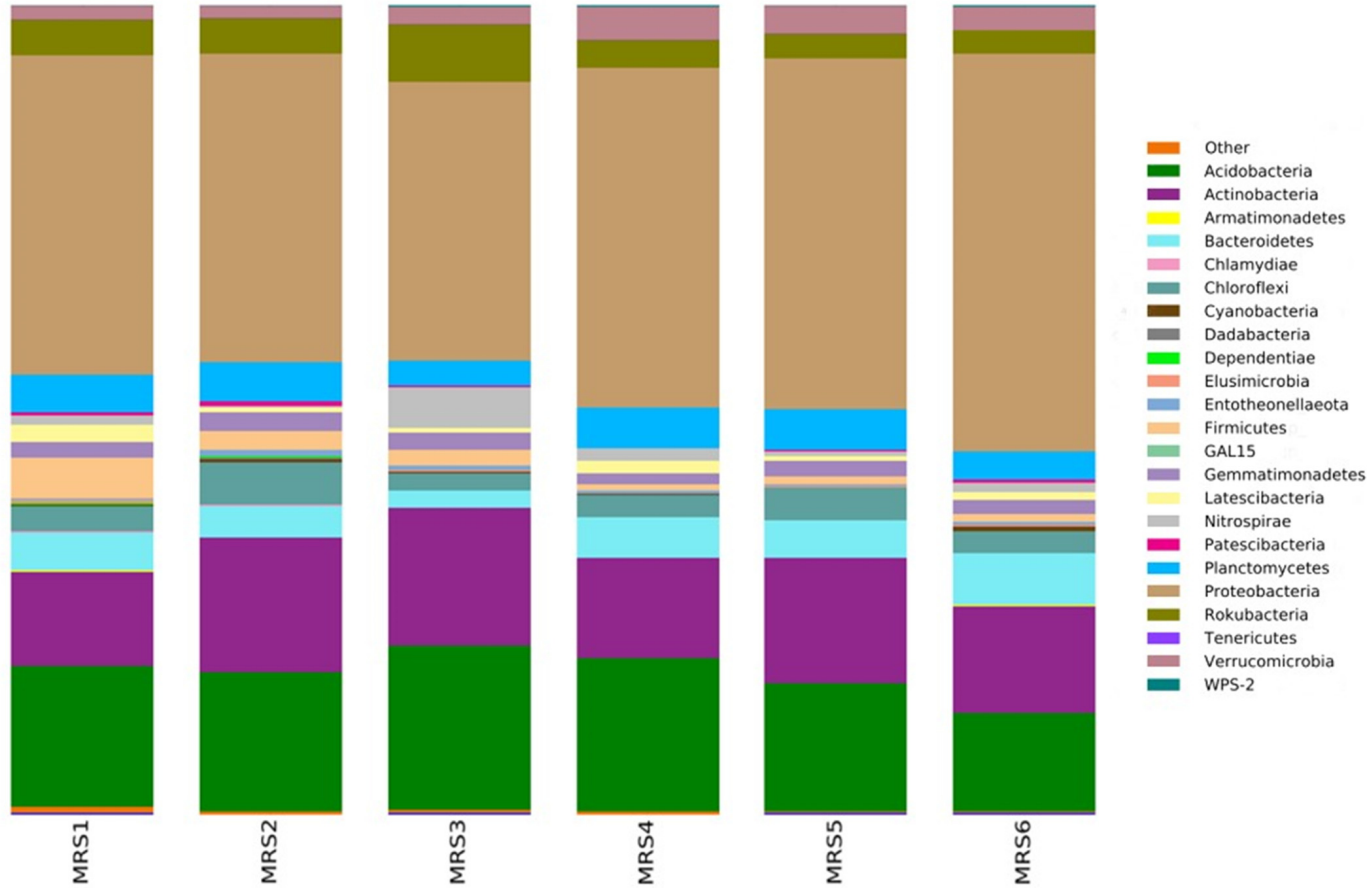
Relative abundance of the top bacterial phyla in each sample obtained from the sequencing data. Each color represents a different phylum.

The total number of reads obtained in this study was 125,158 (Table 1). The OTUs were assigned to 23 bacterial phyla, 72 classes, 165 orders, 248 families, and 357 genera. *Proteobacteria* (41.2%), *Acidobacteria* (17.1%), and *Actinobacteria* (14.4%) were the most represented phyla, and the relative abundance of the top bacterial phyla in each sample is shown in Fig. 1.

**TABLE 1:** Summary description of sample data in this study

Site	Sample	SRA accession no.	No. of sequences	No. of OTUs	Amplicon read length (bp)
Tailing	MRS1	SRR10909566	9,995	957	608
	MRS2	SRR10909565	26,105	1,306	607
	MRS3	SRR10909564	9,199	942	603
Nontailing	MRS4	SRR10909563	28,389	1,213	604
	MRS5	SRR10909562	27,898	1,308	599
	MRS6	SRR10909561	23,572	1,285	568

### Data availability.

The 16S rRNA gene amplicon sequencing data from this study were deposited in the Sequence Read Archive (SRA) of the National Center for Biotechnology Information (NCBI) under the BioProject accession number PRJNA601794 (Table 1).
